# Requirement of Gamma-Carboxyglutamic Acid Modification and Phosphatidylserine Binding for the Activation of Tyro3, Axl, and Mertk Receptors by Growth Arrest-Specific 6

**DOI:** 10.3389/fimmu.2017.01521

**Published:** 2017-11-10

**Authors:** Ke Geng, Sushil Kumar, Stanley G. Kimani, Vladyslav Kholodovych, Canan Kasikara, Kensaku Mizuno, Oleta Sandiford, Pranela Rameshwar, Sergei V. Kotenko, Raymond B. Birge

**Affiliations:** ^1^Department of Microbiology, Biochemistry, and Molecular Genetics, New Jersey Medical School Cancer Center, Rutgers, State University of New Jersey, Newark, New Jersey, United States; ^2^Office of Advanced Research Computing (OARC), Rutgers, State University of New Jersey, Newark, New Jersey, United States; ^3^Department of Pharmacology, Robert Wood Johnson Medical School, Rutgers, State University of New Jersey, Piscataway, NJ, United States; ^4^Department of Biomolecular Sciences, Graduate School of Life Sciences, Tohoku University, Sendai, Miyagi, Japan; ^5^Department of Medicine, Rutgers, State University of New Jersey, Newark, New Jersey, United States

**Keywords:** phosphatidylserine, Tyro3, Axl, and Mertk receptors, growth arrest-specific 6, vitamin K, γ-carboxylation, tumor exosomes

## Abstract

The Tyro3, Axl, and Mertk (TAM) receptors are homologous type I receptor tyrosine kinases that have critical functions in the clearance of apoptotic cells in multicellular organisms. TAMs are activated by their endogenous ligands, growth arrest-specific 6 (Gas6), and protein S (Pros1), that function as bridging molecules between externalized phosphatidylserine (PS) on apoptotic cells and the TAM ectodomains. However, the molecular mechanisms by which Gas6/Pros1 promote TAM activation remains elusive. Using TAM/IFNγR1 reporter cell lines to monitor functional TAM activity, we found that Gas6 activity was exquisitely dependent on vitamin K-mediated γ-carboxylation, whereby replacing vitamin K with anticoagulant warfarin, or by substituting glutamic acid residues involved in PS binding, completely abrogated Gas6 activity as a TAM ligand. Furthermore, using domain and point mutagenesis, Gas6 activity also required both an intact Gla domain and intact EGF-like domains, suggesting these domains function cooperatively in order to achieve TAM activation. Despite the requirement of γ-carboxylation and the functional Gla domain, non-γ-carboxylated Gas6 and Gla deletion/EGF-like domain deletion mutants still retained their ability to bind TAMs and acted as blocking decoy ligands. Finally, we found that distinct sources of PS-positive cells/vesicles (including apoptotic cells, calcium-induced stressed cells, and exosomes) bound Gas6 and acted as cell-derived or exosome-derived ligands to activate TAMs. Taken together, our findings indicate that PS is indispensable for TAM activation by Gas6, and by inference, provides new perspectives on how PS, regulates TAM receptors and efferocytosis.

## Introduction

Tyro3, Axl, and Mertk (TAMs) comprise homologous type I transmembrane receptor tyrosine kinases that are implicated as both oncogenic kinases that drive transformation and tumorigenicity of cancer cells, as well as receptors for the clearance of apoptotic cells and regulate innate immunity and dampen inflammation (1–3). Structurally, TAM receptors have a similar topology and domain organization, whereby their ecto-domains are comprised of dual tandem immunoglobulin-like domains (Ig1 and Ig2) that bind ligand, two fibronectin type III repeats, followed by a transmembrane and intracellular tyrosine kinase domain with a conserved sequence KW(I/L)A(I/L)ES (4–7).

The main ligands for TAMs are two homologous proteins, growth arrest-specific 6 (Gas6) and protein S (Pros1), that are γ-carboxylated by a process that depends on vitamin K. Gas6 and Pros1 also share similar spatial topological homology, including an N-terminus γ-carboxyglutamic acid (Gla) domain, four tandem EGF-like repeats followed by LG1 and LG2 domains, that bind to Ig1 and Ig2 domains of TAM receptors to initiate receptor activation (8, 9). However, despite a high degree of homology between TAMs and their ligands, the ligand-inducible TAM activation follows a biochemical hierarchy whereby (i) Axl is preferentially activated by Gas6 with 100–1,000× higher binding affinity over Mertk and Tyro3 (kd in the nM range), (ii) Tyro3 is preferentially activated by Pros1, and (iii) Merk displays lower sensitivity to both ligand proteins (in the μM range) (10–12). Notably however, both Tyro3 and Mertk become hyperactivated in the presence of phosphatidylserine (PS)-positive liposomes and apoptotic cells, implying that Mertk and Tyro3 may act as “PS sensors” for externalized PS on apoptotic cells and in the tumor microenvironment (13, 14). By utilizing this arrangement, the TAM receptors act as indirect receptors for apoptotic cells, and through their bridging molecules Gas6 and Pros1, are critical for the clearance of apoptotic cells under both homeostatic and stress conditions (15, 16). In mouse models, knockout of TAMs results in inefficient apoptotic cell clearance, and subsequently, in the development of a SLE-like autoimmune condition (17). Single knockout of Mertk partially phenocopies these defects on clearance, whereby resident and bone-marrow derived macrophages fail to engulf apoptotic cells leading to increased circulating inflammatory cytokines and subsequent anti-dsDNA antibodies (18). Therefore, at a functional level, TAMs are thought to have homeostatic roles in higher metazoans that mediate the tolerogenic clearance of apoptotic cells as well as the resolution of inflammation. In other physiological processes, TAMs are also expressed on retinal pigmented epithelial cells (RPEs), Sertoli cells, resident brain microglia where they are involved in the uptake and clearance of rod outer segments, immature spermatogonium, and apoptotic neurons/pruned synapses, respectively, processes that also appear to depend on externalized PS (19–23).

In addition to their homeostatic roles under physiological conditions and in the resolution phase of inflammation, in recent years TAMs have been implicated in human cancers where they have dual roles, first as direct oncogenes expressed on cancer cells to influence proliferation, metastasis, and chemoresistance (3, 24–28), and second as potential checkpoint inhibitors on myeloid cells that induce expression of immunosuppressive cytokines to drive immune escape (26, 29, 30). Therefore, from a targeting therapeutic standpoint, TAMs are intriguing receptor targets (31), since they utilize the same receptor ligand pairs to promote both oncogenic progression and immune escape, and as such, there is great excitement in the field to develop TAM therapeutics. However, despite the importance of TAMs and their ligands in signaling, presently the mechanisms by which Gas6 and Pros1 activate TAMs, and the role that PS plays in this process, is not well understood. Furthermore, no crystal structures or cryo-EM models for Gas6-induced TAM dimerization/activation have been reported. Therefore, how TAMs achieve ligand-dependent dimerization and activation is not clear.

In the present study, we used biochemical, molecular, and reporter cell-based models to study Gas6-mediated activation of TAMs as well as the requirements for PS. Using TAM/IFNγR1 reporter cell lines to monitor functional TAM activity, we found that Gas6 activity was exquisitely dependent on vitamin K-mediated γ-carboxylation, and that replacing vitamin K with anticoagulant warfarin abolished the γ-carboxylation of Gas6, and abrogated activity toward TAM receptors. Furthermore, using domain mutagenesis, we found that Gas6 activity required both an intact Gla domain and intact EGF-like repeats, as mutant Gas6 that carries intact Gla and LG1 and LG2 domains but only lacks EGF-like repeats was also inactive, suggesting these domains are used cooperatively in order to achieve TAM activation, possibly by facilitating dimerization. Using LC/MS/MS to map important γ-carboxylation sites predicted to bind PS, we found that E54/E55 mutants abrogated Gas6 activity, supporting a direct role for PS binding in Gas6-mediated activation of TAMs. In addition, we found that different forms of PS-positive cells and tumor exosomes (comprising important sources of PS in the tumor microenvironment) all recruited Gas6 to their surfaces and acted as cell-based or exosome-based ligands to activate TAMs. Taken together, our findings indicate that PS is indispensable for TAM activation by Gas6, and add new perspectives on how PS impinges on the activation of TAMs.

## Materials and Methods

### Human Gas6-Containing Media (Gas6-CM) and Cell Culture

HEK293 cells were grown in DMEM media supplemented with 10% fetal bovine serum (FBS, Sigma) and 1% penicillin/streptomycin and incubated in 37°C and 5% CO_2_ humidified incubator. To produce wild-type Gas6-CM, when cells reached approximately 60% confluency, pSecTaq-hGas6 (10) was transfected into the cells using LipoD293 transfection reagent (SignaGen). pUcD2SRα-rGas6-Myc plasmids that encode the domain-deleted mutant Gas6 were described previously (32). ΔG, ΔE, and ΔGE represent the mutant Gas6 proteins with Gla domain deleted, EGF-like repeats deleted, and both Gla domain and EGF-like repeats deleted, respectively. The expected molecular weight of these mutant Gas6 proteins is ΔG, 70 kDa; ΔE, 59 kDa; and ΔGE, 49 kDa. These plasmids were transfected into the HEK293 cells with the same method to produce mutant Gas6-CM. 18 h after transfection, the cells were washed twice with PBS and the growth media was replaced with serum-free DMEM media supplemented with 4.4 µM vitamin K (Hospira) or 2 µM warfarin (Sigma) to promote, or abrogate, γ-carboxylation, respectively. The Gas6-CM (Gas6-CM) was collected 72 h later and filtered through 0.22 µm filter. Gas6 concentrations were evaluated using a standard curve against purified recombinant Gas6 (R&D Systems) as previously reported, and unless otherwise stated, approximately 250 nM was used to stimulate TAM receptors in this study (13). Human TAM/IFNγR1 chimeric reporter cells were grown in HAM’s F12 media supplemented with 10% FBS, 2 mM glutamine, and 400 µg/ml of G418 as previously described (10). H1299 and Jurkat cells were grown in RPMI1640 media supplemented with 10% FBS and 1% penicillin/streptomycin. MDA-MB231 and U118 cells were grown in DMEM media supplemented with 10% FBS and 1% penicillin/streptomycin.

### Site-Directed Mutagenesis

Primers for mutagenizing E54 and E55 were designed from QuikChange and purchased from Sigma. The sequences of primers are: Forward 5′-gcgcctttcaggtcttcgacgacgccaagcagg-3′; Reverse 5′-cctgcttggcgtcgtcgaagacctgaaaggcgc-3′. Site-directed mutagenesis was performed by using QuikChange II XL site-directed mutagenesis kit and following the protocol provided within the kit.

### Mass Spectrometric Analysis

The Gas6-CM collected from the transfected HEK293 cells was subjected to SDS-PAGE electrophoresis followed by Coomassie-blue staining. Gel band at approximately 72 kDa of Gas6 was excised and sent for mass spectrometric analysis. In-gel trypsin digestion was performed and the resulting peptides were analyzed by LC-MS/MS on Orbitrap Velos MS. The MS/MS spectra were searched against a Swissprot human database using a local MASCOT search engine (V.2.3).

### Homology Modeling of Gas6/PS Association

A homology model of Gla domain of Gas6 was build based on the available X-ray structure of bovine prothrombin that contained a PS lipid and Ca^2+^ ions, PDB access code is 1NL2. An amino acid sequence of the Gla domain of hGAS6 was obtained from UniProt server (http://www.uniprot.org/), access code Q14393, residues 53–94. After a homology model was created in Molecular Operating Environment (MOE) 2016.08, all CGU (carboxylated glutamic acid residues), a PS lipid and calcium ions were transferred from 1NL2 into the homology model. The model refinement was performed by all atom minimization with Amber10EHT force field in MOE. The second PS lipid was manually docked into the refined model of Gas6 around residue 55 which followed by the second refinement by all atom minimization as described earlier. A structural analysis and a visualization of the interaction network of PS lipids with Gas6 were also completed in MOE (2016.08; Chemical Computing Group ULC, Montreal, QC, Canada, H3A 2R7, 2017).

### Western Blotting

Western blotting was performed as described previously (10). Briefly, Gas6-CM or hTAM/IFNγR1 cell lysate was mixed with 6x sample buffer and subjected to SDS-PAGE gel for electrophoresis, and the antibodies used were as follows: anti-hGas6 monoclonal antibody (R&D Systems), anti-Myc antibody (Cell Signaling Technology), anti-γ-carboxyglutamyl residues monoclonal antibody (Sekisui Diagnostics), anti-STAT1 (pY701) antibody (BD Biosciences), and anti-β-actin antibody (Cell Signaling Technology).

### TAM/IFNγR1 Reporter Cells Stimulation

Human TAM/IFNγR1 cells were seeded in 6-well plate one day prior to stimulation experiment. When the cells reached approximately 90% confluency on the day of experiment, growth media was replaced with serum-free HAM’s F12 medium to starve the cells for 6 h. Gas6-CM or purified Pros1 (isolated from human plasma, purchased from Haematologic Technologies, Inc. HCPS-0090) was added to the reporter cells for 30 min in 37°C incubator. After removing the stimulant, hTAM/IFNγR1 cells were washed with ice-cold PBS, and cellular proteins were extracted by using 1% HNTG lysis buffer (20 mM HEPES, 150 mM NaCl; Triton X100; 10% glycerol; 1 mM phenylmethylsulphonyfluoride; 1 mM sodium vanadate; 0.1 mM sodium molybdate; and 20 µg/ml aprotinin). Protein concentration of cell lysate was determined by Bradford assay using Protein Assay Reagent (Bio-Rad). Same amount of protein from lysate was subjected to SDS-PAGE gel for electrophoresis, and the activation of hTAM/IFNγR1 chimeric receptors was determined by phosphorylated-STAT1 immunoblotting.

For TAM/IFNγR1 cells stimulation by PS positive cells equivalent numbers of apoptotic (UV-treated) or calcium-stressed cells (calcium ionophore A23187-treated) were mixed with Gas6-CM or Pros1 and incubated at room temperature for 30 min first, then the mixture was added to the starved reporter cells for another 30 min at 37°C to trigger receptor activation. To induce PS externalization on native hAxl/IFNγR1 cell lines, the cells were first treated with calcium ionophore A23187, at concentrations of 1, 5, or 10 µM, for 15 min at 37°C. Conversely, to block the externalized PS, PS targeting antibody PGN635 (a gift from Peregrine Pharmaceuticals) was added to the serum-free HAM’s F12 medium at concentrations of 100 and 200 µg/ml and then incubated with the hAxl/IFNγR1 reporter cells for 15 min at 37°C. The antibody-containing solution was washed off by PBS, and Gas6-CM was added to activate the hAxl/IFNγR1 reporter cells.

### PS Externalization

To induce PS externalization by UV-mediated apoptosis, H1299, Jurkat or MDA-MB231 cells were radiated by UV (CL-1000 Ultra Violet Crosslinker, UVP) for 5 min (25,000 µJ/cm^2^) to activate apoptosis pathways. Then the cells were kept in serum-free medium for 2–3 h at 37°C. To induce PS externalization under calcium stress, H1299, Jurkat, and MDA-MB231 cells were detached from plates, washed and treated with 10 µM calcium ionophore A23187 in Ca^2+^/Mg^2+^ free PBS for 15 min at 37°C. The apoptotic or stressed cells were stained with FITC-Annexin V apoptosis detection kit with Propidium Iodide (Biolegend) to assess the extent of PS exposure by flow cytometry following the product’s protocol.

### Exosome Purification

Cell-free media from MDA-MB-231 were collected for exosome isolation using two methods: ExoQuick–TC Exosome Precipitation Solution (System Biosciences) or serial ultracentrifugation at 100,000 *g* for 80 min as previously described (33, 34). The pellets from both methods were washed with PBS and then resuspended in 1 mL of PBS. The samples were diluted at 1/200 and studied by NanoSight Range (Malvern, Westborough, MA, USA). In six different analyses the average particle size was 90 nm with 5.35 × 10^10^ particles/ml.

### PS Staining on the Surface of Exosomes

Exosomes purified from MDA-MB-231 cells were first isolated by using exosome-human CD63 isolation reagent following manufacturer’s instruction (Invitrogen Thermofisher). Briefly, the exosomes were incubated with the Dynabeads overnight at 4°C. After this, the beads were washed twice with PBS, with the aid of a magnetic stand. The bound exosomes were stained with FITC-Annexin V using the Apoptosis Detection Kit (BioLegend) to assess PS distribution on the surface by flow cytometry.

### Gas6 Binding by Flow Cytometry

Growth arrest-specific 6 binding assays were performed following the method described by Dransfield et al. (35). Briefly, calcium-stressed H1299 cells were firstly prepared as described above and then washed once and re-suspended in washing buffer (PBS with 1 mM CaCl_2_ and 1 mM MgCl_2_). 50 nM of recombinant Gas6 (R&D Systems) was added to the cell and incubated in rotation at room temperature for 30 min. The cells were then washed with washing buffer and re-suspended in washing buffer with diluted (1:100) primary Gas6 antibody (Abcam, Ab136249) and incubated in rotation at 4°C for 40 min. After this, the cells were washed twice using washing buffer to remove unbound antibody, and an Alexa Fluor 647-conjugated secondary antibody (Rabbit IgG, Thermo Fisher) diluted (1:500) in washing buffer was added to the cells for 30 min incubation at 4°C in dark. The cells were washed again to remove unbound antibody, and Gas6 binding to the cell surface was assessed by flow cytometry.

To assess Gas6 binding to Axl receptor, hAxl/IFNγR1 cells were detached from the plate and washed twice with PBS that contains 5 mM EDTA to eliminate natively-bound proteins. The cells were then centrifuged and resuspended in the Myc-tagged wild-type or mutant Gas6-CM for 30 min in rotation at room temperature. The cells were then washed once with washing buffer and resuspended in diluted (1:100) anti-Myc-PE antibody (Cell Signaling Technology) for 40 min at 4°C in dark. The cells were washed again to remove unbound antibody, and Gas6 binding to Axl receptors was assessed by flow cytometry.

### Statistical Analysis

Student’s *t*-tests or two-way ANOVA followed by post/hoc tests were performed to analyze statistical differences between groups using GraphPad Prism software. *p* Values lesser than 0.05 were considered significant (*p < 0.05; **p < 0.005; ***p < 0.001; ****p < 0.0001).

## Results

### γ-Carboxylation of the Gas6 Gla Domain Is Required for Gas6 Activity

In a previous study, we developed TAM/IFNγR1 chimeric receptor CHO reporter cell lines, whereby the extracellular and trans-membrane domains of each human and mouse TAM receptor were fused in frame to the intracellular domain of IFNγR1 to investigate the molecular mechanisms of TAM activation by endogenous ligands Gas6 and Pros1 (10). Using these reporters, and subsequently validating their utility with native TAM receptors, we reported that TAMs have differential specificities for Gas6 and Pros1, as well as differential affinities as PS sensing receptors to stimulate TAM activity and induce efferocytosis (13). Here, we extended these studies to investigate the requisite role of PS with respect to Gas6-mediated TAM activation, as well as inquiring whether PS externalization on apoptotic cells, calcium-stressed cells, or tumor exosomes have different capabilities to activate TAMs.

**Figure 1:**
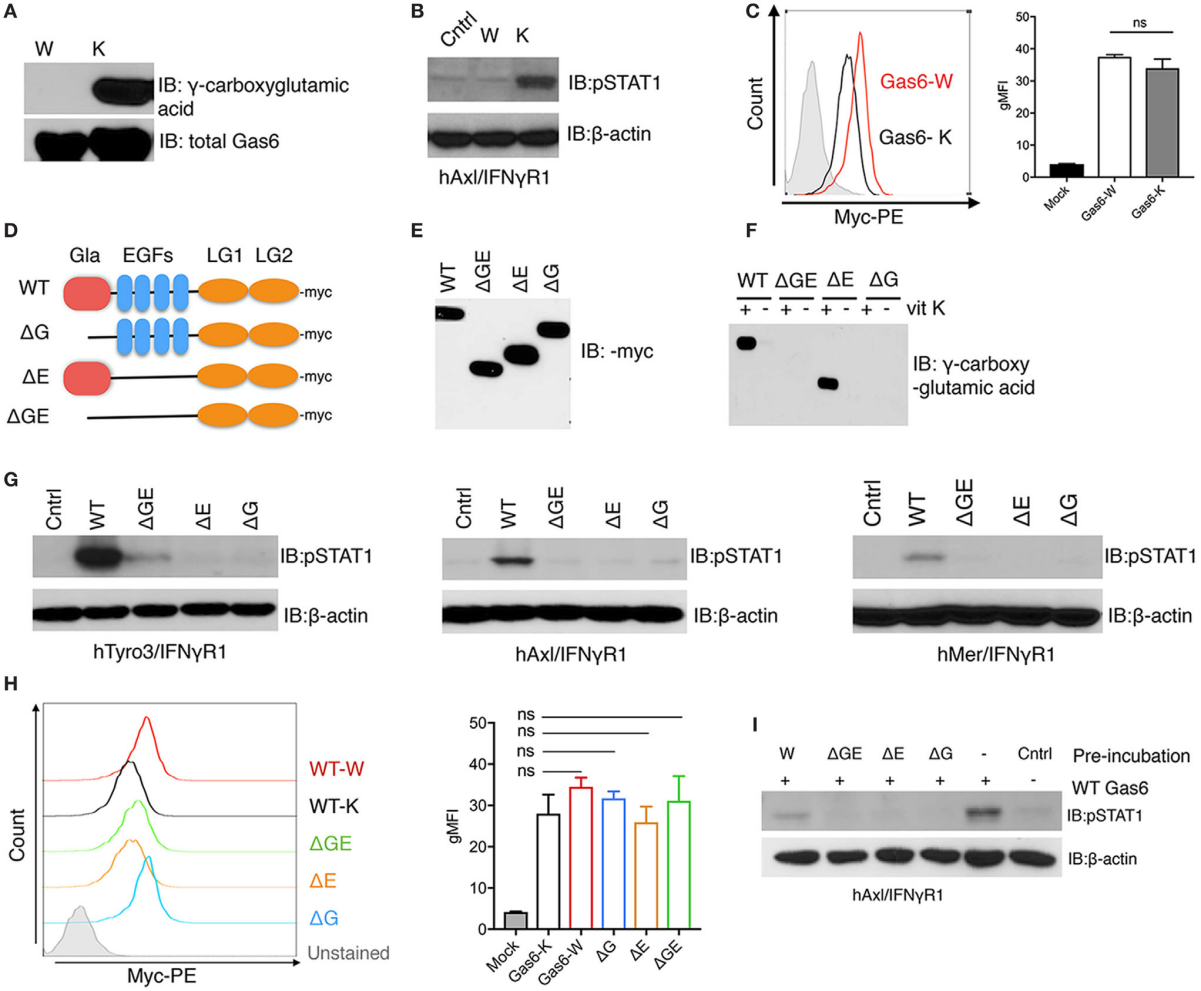
γ-Carboxylation is required for growth arrest-specific 6 (Gas6) activity. **(A)** HEK293 cells were transfected with pSecTaq-hGas6 plasmid (10) to collect Gas6-containing media (CM). Serum-free culture media was supplemented with either 2 µM warfarin (W) or 4.4 µM vitamin K (K) to abolish or induce γ-carboxylation of Gas6. The conditioned media was subjected to SDS-PAGE gel to assess total Gas6 and γ-carboxylation level by Gas6 and γ-carboxylglutamic acid immunoblotting. **(B)** hAxl/IFNγR1 reporter cells were stimulated by warfarin-treated Gas6-CM (W) or vitamin K-treated Gas6-CM (K). The activation of Axl receptor was assessed by pSTAT1 immunoblotting. **(C)** hAxl/IFNγR1 cells were incubated with warfarin-treated Gas6 (Gas6-W, red) conditioned media or vitamin K-treated Gas6 (Gas6-K, black) conditioned media. The binding of Gas6 to hAxl/IFNγR1 receptors was assessed in flow cytometry by using anti-Myc-PE antibody (left), with geometric MFI quantification from independent experiments (*n* = 3) in the right panel. Error bar, SEM. n.s., not significant. **(D)** Schematic structure of Myc-tagged mutant Gas6 proteins. WT, wild-type Gas6; ΔG, Gas6 that lacks Gla domain; ΔE, Gas6 that lacks EGF repeats; ΔGE, Gas6 that lacks both Gla domain and EGF repeats. **(E)** HEK293 cells were transfected with the pUcD2SRα-rGas6-Myc plasmid (32) in the presence or absence of vitamin K. The expression level of the Gas6 proteins in the conditioned media was assessed by Myc-immunoblotting. **(F)** The γ-carboxylation level of the Gas6 proteins was assessed by γ-carboxylglutamic acid immunoblotting. **(G)** Human TAM/IFNγR1 reporter cells were stimulated by wild-type or mutant Gas6-CM, and the activations of TAM receptors were assessed by pSTAT1 immunoblotting. **(H)** The binding of different (warfarin or vitamin K-treated wild-type, or mutant) Gas6 proteins to hAxl/IFNγR1 cells was assessed by flow cytometry using anti-Myc-PE antibody (left), with MFI quantification from independent experiments (*n* = 3) in the right panel. Error bar, SEM, n.s., not significant. **(I)** Warfarin-treated (W) or mutant Gas6 was added to hAxl/IFNγR1 cells in culture dish for 30 min. The cells were then washed by PBS and wild-type Gas6 with fully γ-carboxylation was added to the cells. The activation of Axl was assessed by pSTAT1 immunoblotting. The Western blotting results are representative results from at least three independent experiments.

To examine the requirement of γ-carboxylation and Gas6 activity toward TAM receptors, we generated recombinant non-γ-carboxylated Gas6 by culturing Gas6 expressing HEK293 cells in the presence of warfarin, an anticoagulant that blocks vitamin K epoxide reductase, an enzyme that reduces vitamin K to its active form, thereby blocking an essential step in vitamin K-dependent γ-carboxylation (36). As shown in Figure 1A, when we assessed non-γ-carboxylated versus fully γ-carboxylated Gas6 proteins (produced in the presence of exogenous vitamin K), the warfarin-treated Gas6 showed no detectable γ-carboxylation compared with its vitamin K-treated counterpart, and this was confirmed by LC/MS/MS (data not shown). Moreover, while overall production yields of non-γ-carboxylated and γ-carboxylated Gas6 were similar (Figure 1A. lower panel), only γ-carboxylated Gas6 activated hAxl/IFNγR1 reporter cells and the non-γ-carboxylated Gas6 was inactive under these conditions [compare warfarin (W) versus vitamin K (K) in Figure 1B]. Interestingly, however, while only γ-carboxylated Gas6 was effective to activate hAxl-IFNγR1 cells, both non-carboxylated (Gas6-W) and γ-carboxylated (Gas6-K) forms of Gas6 are capable of binding to the hAxl-IFNγR1 cells with similar efficacy (i.e., geometric mean intensity) (Figure 1C), suggesting that while γ-carboxylation is not required for Axl binding, it appears to be critical to induce a conformational change following Gas6 binding to Axl, presumably in order to achieve dimerization and postreceptor signaling.

### Structure/Activity Analysis of Gas6 by Domain Mutagenesis

To better understand why N-terminal γ-carboxylation is required for TAM activation (which is mediated *via* the binding of the carboxyl-terminal LG1 and LG2 domains to TAMs), we performed structure activity experiments on Gas6 (for TAM activation) using a series of Gas6 domain deletion mutants (Figure 1D). Previous studies, for example, predicted that non-γ-carboxylated Gas6 might form an intramolecular inhibitory structure to block LG domains, that becomes released by γ-carboxylation of the Gla domain to activate Gas6 (32). To explore this idea, we expressed Myc-tagged domain mutant recombinant Gas6 proteins (Figure 1D), either in the presence of warfarin or vitamin K to generate secreted recombinant mutant proteins expressed in similar amounts (Figure 1E). As expected, only WT Gas6 and ΔE mutant Gas6 (that deletes the 4 tandem EGF-like domains) showed vitamin K-dependent γ-carboxylation (Figure 1F, lanes 1 and 5), which were subsequently blocked in Gas6-producing cells treated with warfarin (Figure 1F, lanes 2 and 6). Interestingly, however, when Gas6 domain mutants were tested on the TAM/IFNγR1 cell lines (Tyro3/IFNγR1, Axl/IFNγR1, and Mertk/IFNγR1 lines), none of the mutant Gas6 proteins (ΔG, ΔE, and ΔGE) showed detectable activity for TAM receptors compared to WT Gas6 (Figure 1G). Notably, the lack of activity of the ΔE mutant Gas6 (that contains Gla and LG1/LG2 domains) implies that the 4 tandem EGF-like repeats are also required for Gas6-mediated TAM activation, a domain region of Gas6 that has not previously been implicated in regulating the function of Gas6.

To explore whether Myc-Gas6 mutants retained ability to bind to the TAM receptors (and are functional proteins), we incubated each of the aforementioned proteins with hAxl/IFNγR1 reporter cells. All of the aforementioned proteins bound TAMs as evident by flow cytometry (Figure 1H) with similar efficacy (i.e., geometric mean intensity) but could not dimerize/activate the TAMs (Figure 1G). Indeed, when mutant proteins were first incubated with hAxl/IFNγR1 cells, and subsequently cells were treated with WT Gas6, prior exposure to inactive mutant proteins blocked subsequent Gas6 activity, suggesting they may act as dominant negative or ligand traps to block TAM function (Figure 1I). Indeed, these data are consistent with previous reports that warfarin can block TAM signaling in cancer models by producing inactive TAM ligands (29).

### Mapping Gas6 γ-Carboxylation by LC-MS/MS

**Figure 2:**
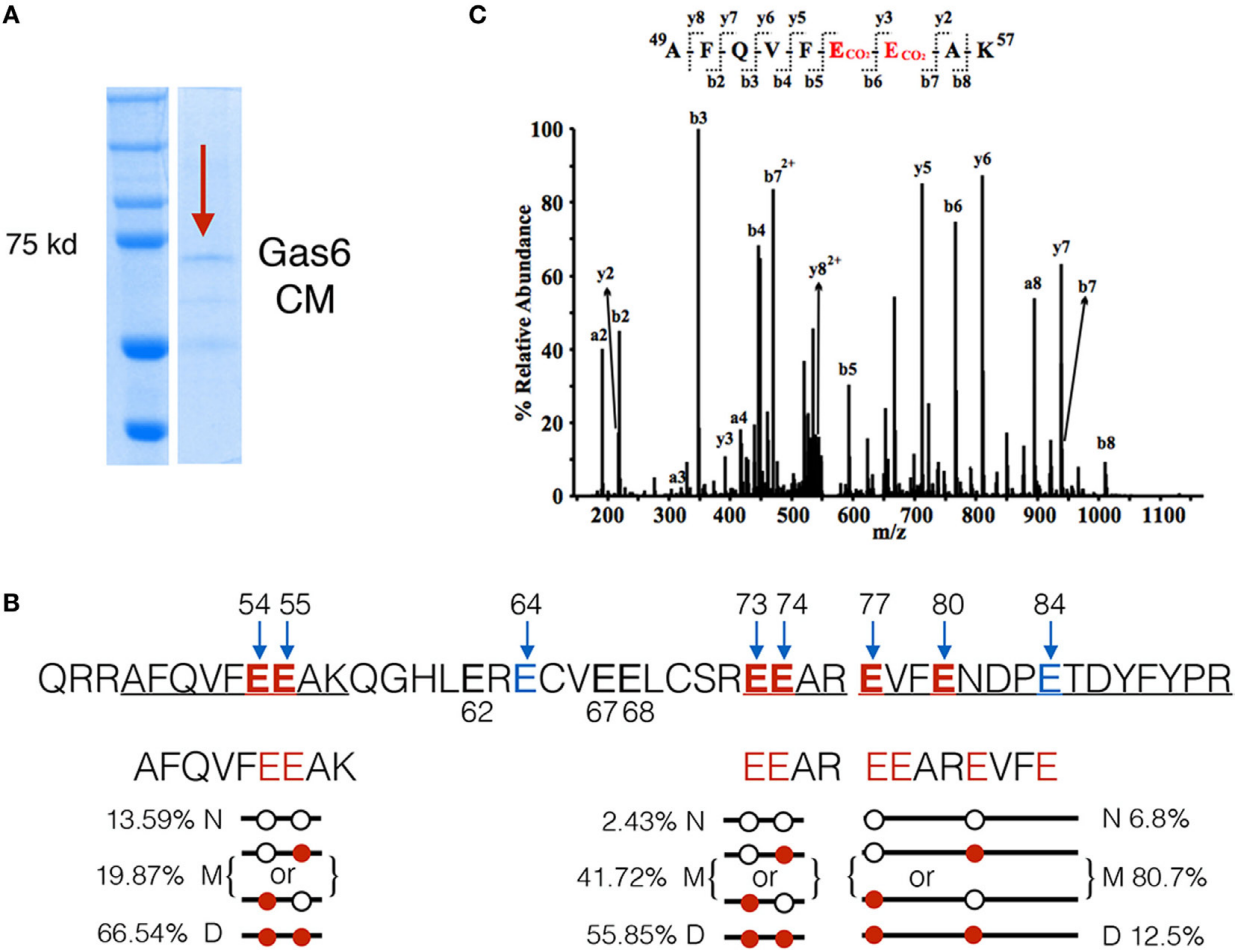
LC-MS/MS analysis of γ-carboxylation sites in Gla domain of growth arrest-specific 6 (Gas6). **(A)** Gas6-containing media (Gas6-CM) was subjected to SDS-PAGE electrophoresis, and the band at 72 kDa was excised and subjected to LC-MS/MS analysis. **(B)** In the Gla domain of Gas6, glutamic acids 54, 55, 64, 73, 74, 77, 80, and 84 were identified as γ-carboxylated by LC-MS/MS. Among them, three independent carboxylated peptides, ^49^AFQVFEEAK^57^, ^73^EEAR^76^, and ^77^EVFENDPETDYFYPR^91^, were identified with glutamic acid residues shown in red. Each peptide was found in three forms: non-γ-carboxylated (N), single carboxylated at one of the E residues (M), or dually carboxylated at both E residues **(D)**. The frequencies of each form of the peptides were listed. **(C)** Representative MS/MS spectrum of a doubly-charged ion (*m/z* 578.76) is corresponding to the peptide sequence of ^49^AFQVFEEAK^57^ with carboxylation modification at E54 and E55. The observed *y-* and *b-*ion series confirmed the peptide sequence and modification.

The above mentioned experiments employing a series of Gas6 domain mutants implicated γ-carboxylation as an essential feature for Gas6-mediated activation of TAMs. To investigate the relationship between γ-carboxylation and binding to PS in more detail, we performed LC-MS/MS analysis on hGas6, prepared from vitamin K-treated Gas6-producing cells (Figure 2A). After SDS-PAGE electrophoresis, the Gas6 band at ~72 kDa was excised, and peptide bands were assessed for γ-carboxylation (i.e., increased mass of 44 Da). Under these conditions, (whereby Gas6 is functionally active; Figure 1), we isolated three peptides from the trypsin-digested protein for stoichiometry analysis, and found a complex pattern of γ-carboxylated glutamic acids in the active species of Gas6. A representative MS/MS spectrum of the γ-carboxylated peptide ^49^AFQVFEEAK^57^ was shown in Figure 2C. For example, in the peptide ^49^AFQVFEEAK^57^, E54 and E55 were found either dually γ-carboxylated at a frequency of 66.5%, or single γ-carboxlated at one or the other glutamic acid at a frequency of 19.87%, or non-carboxylated at a frequency of only 13.6%. Similarly, for peptide ^73^EEAR^76^, E73 and E74 were either dually γ-carboxylated at a frequency of 55.85%, or single γ-carboxylated at one or the other residue at 41.72%, or non-carboxylated at a frequency of 2.43%, while in a third γ-carboxylated peptide ^77^EVFENDPETDYFYPR^91^, the frequency of dually, single, and non-γ-carboxylation at E77 and E80 were found to be 12.5, 80.7, and 6.8%, respectively (Figure 2B). Other residues at lower stoichiometry included E64, E67, and E84. Together, these data suggest that γ-carboxylation of Gas6 is highly dynamic, likely to fine-tune Gas6 activity *via* the activity of enzymes that are required for this post-translational modification, such as gamma-glutamyl carboxylase (GGCX) and vitamin K epoxide reductase complex I (VCOR1).

### Model for Interaction between Gas6-Gla Domain and PS Based on the PDB Structure of Bovine Prothrombin

**Figure 3:**
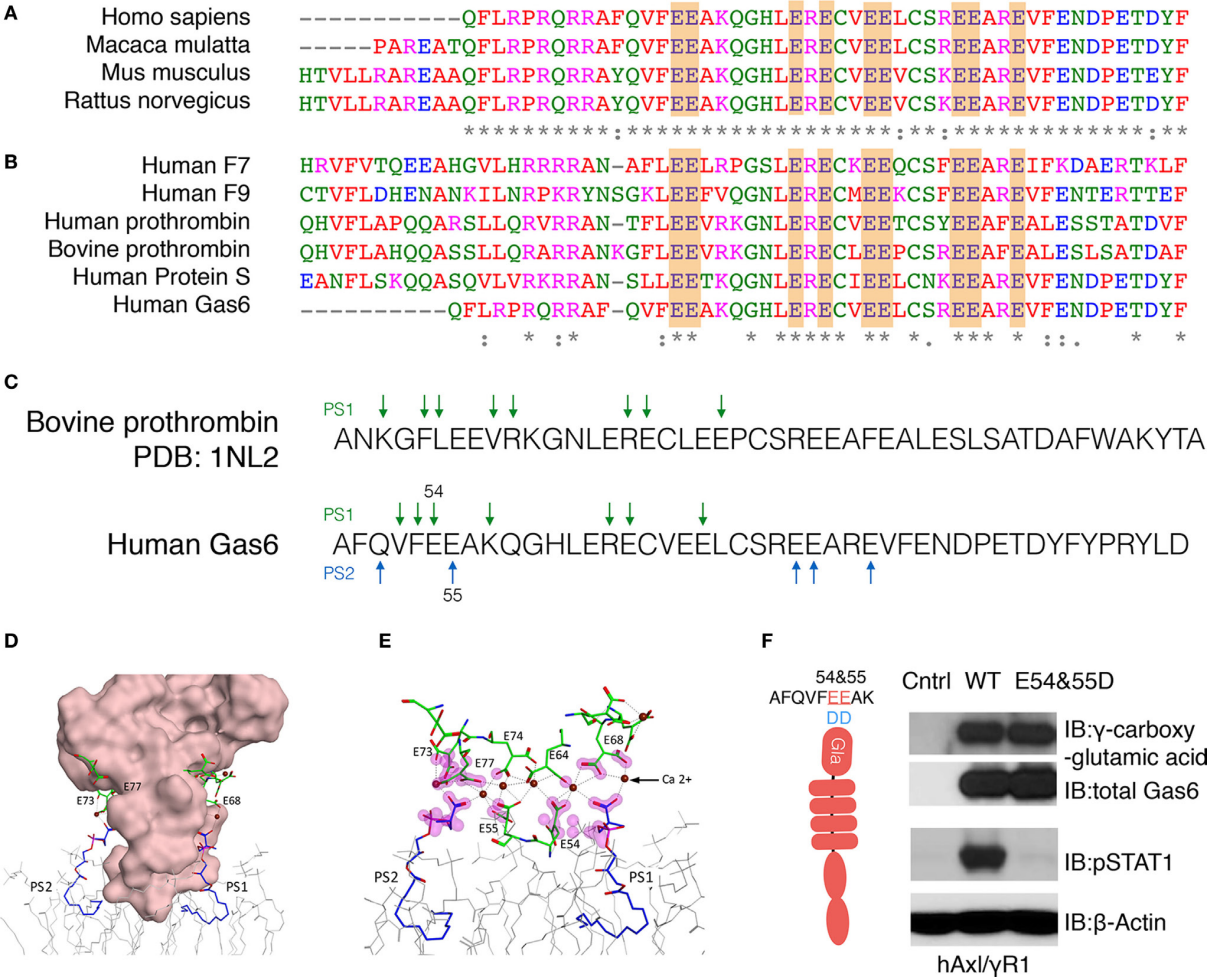
Homology modeling for the association between Gla domain of growth arrest-specific 6 (Gas6) and phosphatidylserine (PS). **(A)** The sequences of Gla domain of Gas6 proteins between species and **(B)** the sequences of Gla domain of different human vitamin K-dependent proteins were pairwise aligned with conserved glutamic acids highlighted in orange. **(C)** Homology model of Gla domain of Gas6 is based on Gla domain of bovine prothrombin PDB: 1NL2. The amino acids that may provide binding sites for PS were pointed out by green arrows. In human Gas6, two PS molecules (PS1 and PS2) are predicted to be associated with Gla domain from two symmetrical interfaces of the protein; those amino acids were pointed out by green and blue arrows separately. **(D)** Molecular surface of Gla domain of Gas6 was shown in pink, and the γ-carboxylated E residues shown in green, associated with calcium ions as brown balls, two PS molecules are shown in blue and POC lipids as gray wire models. **(E)** Possible contacts between PS and E residues are highlighted with magenta clouds. **(F)** Point mutations were induced to switch 54 and 55 glutamic acids (E) to aspartic acids (D). HEK293 cells were transfected with the mutant Gas6 plasmid, and the γ-carboxylation and expression of wild-type or E54 and 55D Gas6 proteins were assessed by γ-carboxylglutamic acid or Gas6 immunoblotting (upper panel). hAxl/IFNγR1 reporter cells were stimulated by wild-type or E54 and 55D Gas6, and the activation of Axl was assessed by pSTAT1 immunoblotting (lower panel). The Western Blotting results are representative results from at least three independent experiments.

Having identified a complex pattern in the γ-carboxylation of active Gas6, including residues E54, E55, E64, E73, E74, E77, E80, and E84 we aligned hGas6 across species, as well as to other γ-carboxylated clotting factors that belong to the vitamin K-dependent proteins family, that require γ-carboxylated in their Gla domains to bind PS exposed on activated platelets (37). As shown in Figures 3A,B, the arrangement of glutamic acid motifs in Gla domain of Gas6 is well-conserved across species, and pairwise analysis showed considerable conservation among members of Gla-containing proteins in human such as F7, F9, thrombin, Pros1, Gas6, and bovine prothrombin, that latter of which there is a derived crystal structure in the presence of PS (38). Indeed, based on the available structure and the high degree of homology between bovine Prothrombin and human Gas6 (Figure 3C), we generated homology models of hGas6 in complex with PS.

From these models and the experimental-derived constellation of γ-carboxylated glutamic residues identified in the Gla domain of Gas6 by LC-MS/MS, a synergy model is predicted whereby two or more PS head-groups can interact symmetrically with the Gla domain. In these models, one molecule of PS (PS1) is predicted to interact with E54 and E64 on one surface, while a second molecule of PS (PS2) is predicted to interact with E55, E73, and E77 on the opposite surface of the Gla domain (Figures 3D,E). Indeed, since the E54/E55 motif are located at the base of the Gla domain that makes a direct contact with phospholipids, and therefore predicted to bind PS directly, we generated a Gas6 E54/E55 double mutant (Figure 3F). As indicated, this mutant abrogated Gas6-mediated activation of hAxl/IFNγR1 cells, even though E54D/E55D retained almost complete net γ-carboxylation levels, as evident from Western blotting with an anti-γ-carboxyglutamic acid (Gla) specific mAb (Figure 3F). These data suggest that critical PS interacting glutamic acid residues in Gas6, when substituted, abrogate ligand-binding activity of Gas6, further supporting this idea that direct PS binding is indispensable for Gas6-mediated activation of TAMs.

### PS-Positive Cells Opsonize Gas6 and Stimulate TAMs

**Figure 4:**
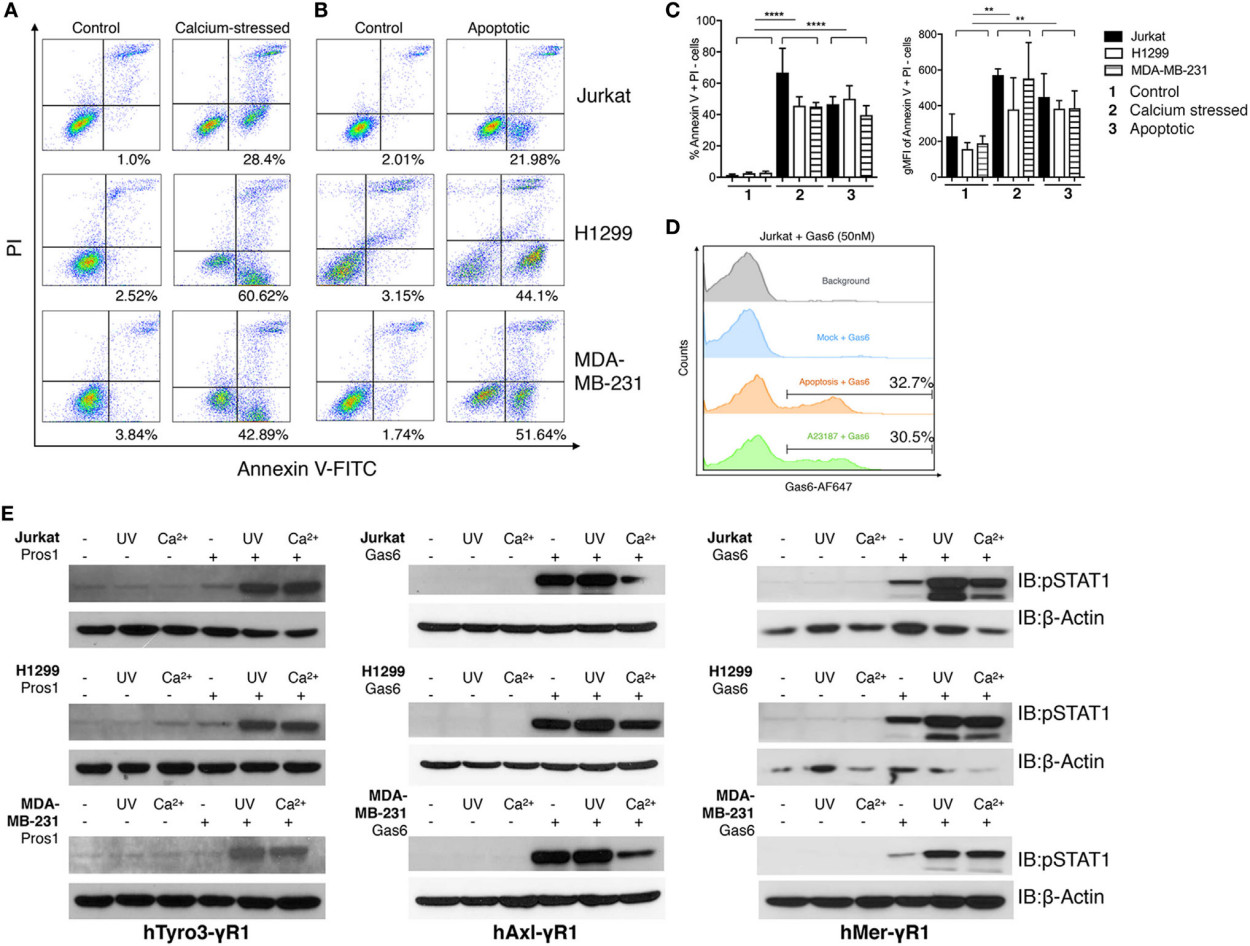
Phosphatidylserine (PS)-positive apoptotic and calcium-stressed cells differentially affect Tyro3, Axl, and Mertk (TAMs) activation by growth arrest-specific 6 (Gas6) or protein S (Pros1). **(A)** Jurkat, H1299, and MDA-MB231 cells (top to bottom) were treated with calcium ionophore A23187 (10 μM) for 15 min in 37°C and then stained with FITC-Annexin V and PI to assess PS externalization. **(B)** Jurkat, H1299, and MDA-MB231 cells (top to bottom) were treated with UV radiation and then stained with FITC-Annexin V and PI to assess PS externalization. **(C)** Percentage (left) and MFI (right) quantification of the Annexin V^+^/PI^−^ cells that were induced by calcium stress or UV radiation from independent experiments (*n* = 3). Error bar, SEM, **p < 0.005, ****p < 0.0001. **(D)** Recombinant Gas6 protein at concentration of 50 nM was added to 1.0 × 10^6^ apoptotic or calcium-stressed Jurkat cells and subsequently stained with anti-hGas6 primary and antirabbit Alexa Fluor 647 secondary antibody to assess Gas6-cell surface association by flow cytometry. Background (gray): Jurkat cells without treatment were stained with primary and secondary antibodies; Mock + Gas6 (blue): Jurkat cells without treatment were incubated with 50 nM Gas6 and then stained with primary and secondary antibodies. **(E)** Three modes (resting, calcium-stressed and apoptotic) of Jurkat, H1299 and MDA-MB231 cells were mixed with Gas6-containing media (Gas6-CM, Gas6 concentration estimated as 250 nM) or Pros1 (100 nM) to stimulate hTAM/IFNγR1 reporter cells. The activation of TAM receptors was assessed by pSTAT1 immunoblotting. The Western blotting results are representative results from at least three independent experiments.

Given the essential role for PS for the Gas6-mediated activation of TAMs, we next examined physiological sources of PS, and how they influence Gas6-mediated activation of TAMs (Figure 4). It is well known that PS is predominantly distributed on the inner leaflet of the plasma membrane, but can be externalized (i) during apoptosis, (ii) during cell stress (particularly calcium induced stress), and (iii) on exosomes derived from tumor cells (tumor exosomes) (39, 40). Over the past several years, molecular mechanisms for PS externalization have been described, including the identification of scramblases and mechanisms of PS externalization under the aforementioned conditions. For example, during apoptosis, PS is externalized *via* activation of a caspase 3/caspase 7-activated scramblase called Xkr8 (41), while PS is externalized in calcium-stress cells by a calcium-activated scramblase called TMEM16F (42).

In order to compare physiological sources of externalized PS, including PS externalized following calcium ionophore treatment (stress) versus PS externalized following UV irradiation (apoptosis), and how these cells impinge on Gas6-mediated TAM activation, we induced PS externalization on distinct cancer cell lines, including Jurkat T cell leukemia cells, MDA-MB-231 breast cancer cells, and H1299 lung cancer cells, and tested their ability to (i) recruit soluble recombinant Gas6 to their surface and (ii) to activate, as cell-based ligands, TAM/IFNγR1 reporter lines *via* cell to cell interactions (Figure 4).

As shown in Figure 4A, When Jurkat cells, MDA-MB-231 cells, or H1299 cells were transiently exposed to calcium ionophore A23187 for 15 min as an acute non-apoptotic stressor, all three cell lines displayed clear PS externalization (as evident from FITC-Annexin V positive/PI-negative staining). In an independent experiment, these cells also expose PS on their surface due to the UV radiation-induced apoptosis (Figure 4B). Quantifications using geometric mean staining of annexin V-positive cells revealed similar PS externalization under both conditions (Figure 4C).

Next, to assess whether PS positive cells induced by stress versus apoptosis recruited Gas6, cells were normalized for the extent of PS exposure, and subsequently incubated cells with 50 nM recombinant Gas6. Under these conditions, both cell populations recruited Gas6 to a similar amount (~30%) as evident by flow cytometric analysis (Figure 4D). Subsequently, aforementioned PS positive cells from Figure 4A were normalized for PS (after flow cytometry), incubated with Pros1 or Gas6-CM, and further added to the TAM/IFNγR1 reporter lines as opsonized cell-based ligands. As indicated, both apoptotic cells and calcium stressed cells profoundly enhanced Mertk and Tyro3 activation by Gas6 and Pros1, respectively, compared with receptor activation induced by Gas6 or Pros1 alone. Axl activation by Gas6, on the other hand, was not further enhanced in the presence of apoptotic cells and decreased by calcium stressed cells in some cases (Figure 4E). These data support our previous models and suggest that among TAMs, mainly Tyro3 and Mertk act as PS sensors to amplify PS signaling from stress-activated or apoptotic cells.

### PS-Positive Tumor Exosomes Opsonize Gas6 and Stimulate TAMs

**Figure 5:**
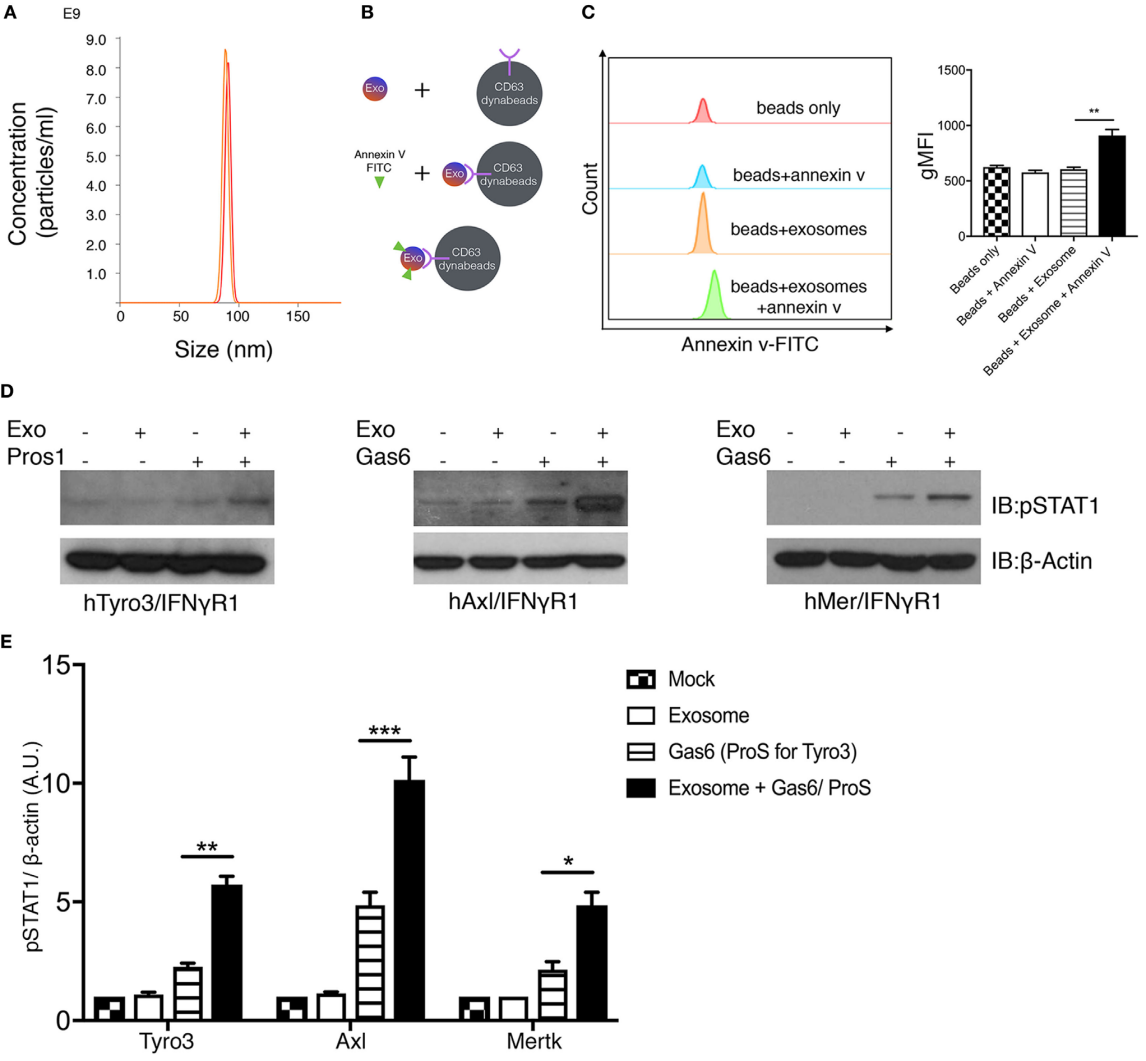
Phosphatidylserine (PS)-positive tumor exosomes enhance Tyro3, Axl, and Mertk (TAMs) activation by growth arrest-specific 6 (Gas6) or protein S (Pros1). **(A)** Tumor-derived exosomes were purified from MDA-MB-231 cell’s culture media, and the size and concentration of the exosomes were analyzed by NanoSight Tracking Analysis. **(B)** Schematic procedure of PS staining of exosomes using CD63-Dynabeads. Exosomes isolated from MDA-MB231 cells were incubated with CD63-Dynabeads and then stained with FITC-Annexin V to assess PS exposure on the surface of exosomes. The experiment results are shown in **(C)** in which the samples from top to bottom are: beads alone (red), beads + FITC-Annexin V (blue), beads + exosomes (orange), beads + exosomes + FITC-Annexin V (green). The MFI quantification is shown in the right panel from independent experiments (*n* = 3). Error bar, SEM, **p < 0.005. **(D)** Exosomes that were isolated from MDA-MB231 cells were mixed with Gas6-containing media (Gas6-CM) or Pros1 (100 nM) to stimulate hTAM/IFNγR1 reporter cells. The activation of TAM receptors was assessed by pSTAT1 immunoblotting. **(E)** Densitometric quantification of hTAM/IFNγR1 reporter cells’ activation by exosomes (pSTAT1) normalized to β-actin from independent experiments (*n* = 3). Error bar, SEM. *p < 0.05, **p < 0.005, ***p < 0.001.

Having established that apoptotic and calcium-mediated stressed cells, *via* externalized PS, could act as cell-based ligands to activate TAMs in the presence of Gas6/Pros1, we next explored whether PS-positive tumor exosomes could also activate TAMs (Figure 5). To address this, tumor-derived exosomes were first isolated from either cultured MDA-MB-231 cells or U118 glioblastoma cells (data not shown) after which, nanoparticles with diameter in the range of 70–100 nm were purified by ultracentrifugation (Figure 5A). To assess whether the tumor-derived exosomes were positive for PS, we subsequently incubated the vesicles from Figure 5A with Dynabeads conjugated with anti-CD63 antibody, followed FITC-Annexin V staining (Figure 5B). As shown in Figure 5C, only the “exosome + beads” were stained the FITC-Annexin V, indicating PS distribution on the surface of the exosomes. These results revealed that in addition to apoptotic and calcium-stressed cancer cells, tumor exosomes are also PS-positive, in agreement with recent published results of Schroit and colleagues (40). To assess whether Gas6/Pros1-opsonized exosomes could activate TAMs, they were subsequently incubated with the hTAM/IFNγR1 reporter cells. Under these conditions, PS-positive exosomes (opsonized with their TAM ligands, Gas6 or Pros1) reproducibly hyper-activated all three TAM receptors (Figures 5D,E). This pattern of activation of Axl by PS + exosomes is distinct to the Axl activation observed for PS + apoptotic cells and PS + calcium stressed cells (Figure 4E), possibly suggesting different mechanisms of Axl activation by exosomes.

### Cell Intrinsic Role for Gas6 in the Activation of TAMs

**Figure 6:**
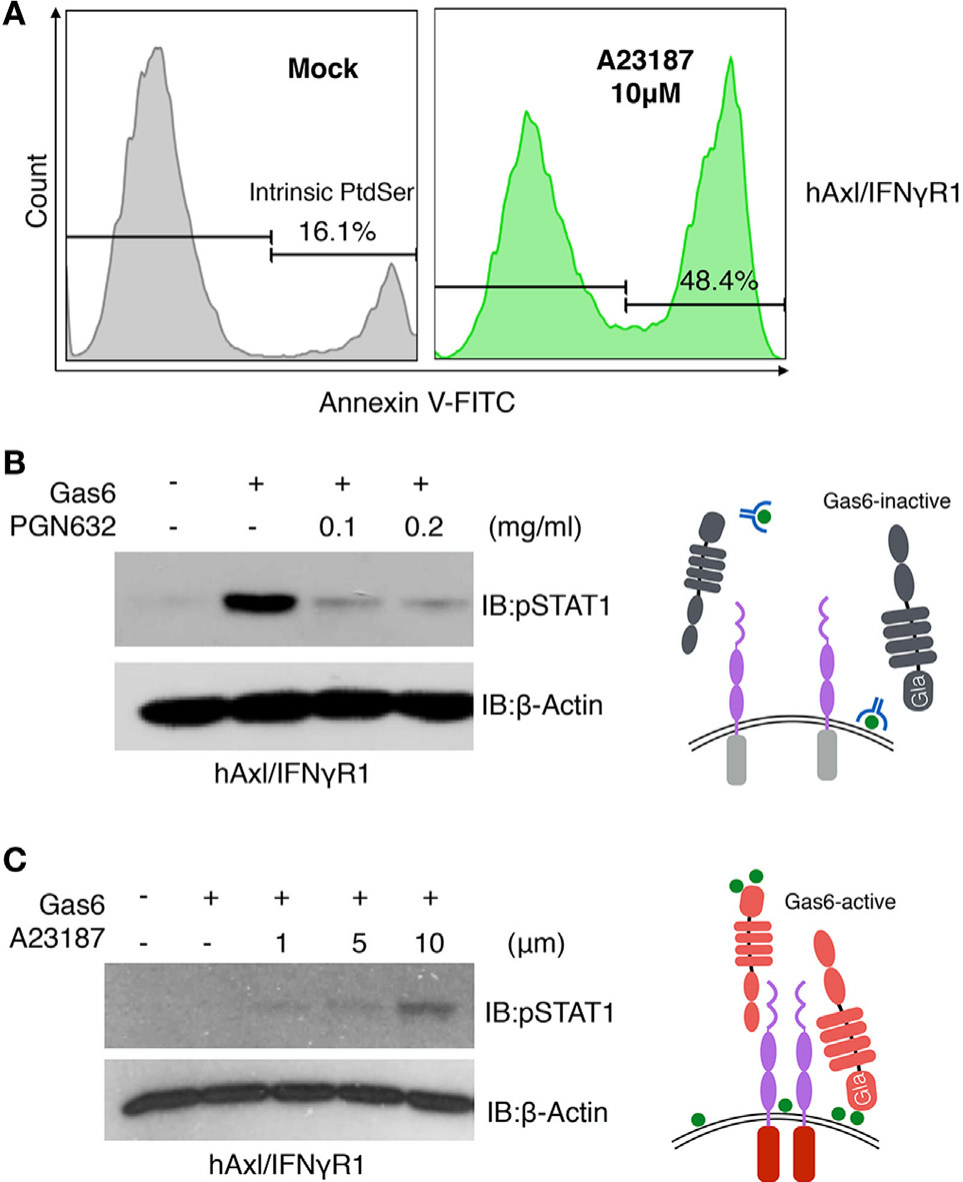
Phosphatidylserine (PS) is involved in growth arrest-specific 6 (Gas6)-Axl activation after calcium-ionophore treatment. **(A)** hAxl/IFNγR1 cells that received no treatment (gray) or 10 µM calcium ionophore A23187 (green) at 37°C for 15 min were stained with FITC-Annexin V to assess PS externalization. **(B)** PGN632, a PS-targeting reagent, was added to hAxl/IFNγR1 reporter cells at two concentrations (0.1 and 0.2 mg/ml) for 20 min. The reporter cells were washed and stimulated by hGas6-CM subsequently. The activation of Axl receptor was assessed by pSTAT1 immunoblotting. Schematic illustration of this experiment is shown in the right panel. **(C)** hAxl/IFNγR1 reporter cells were first treated with calcium ionophore A23187 at concentrations of 1, 5, and 10 µM and then stimulated by hGas6-CM. The activation of Axl receptor was assessed by pSTAT1 immunoblotting. Schematic illustration of this experiment is shown in the right panel. The Western blotting results are representative results from at least three independent experiments.

The aforementioned findings that externalized PS from calcium-activated cells or apoptotic cells could act *in trans* as cell-derived opsonins to activate TAM reporter lines. However, to evaluate whether PS externalization on TAM expressing cells could also act intrinsically, i.e., *in cis, to* activate TAMs by an autocrine mechanism (Figure 6), we treated the hAxl/IFNγR1 cell line with 10 µM calcium ionophore A23187 to achieve PS externalization. As shown in Figure 6A, 16.1% of the cells were stained with FITC-Annexin V, indicating the native existence of PS-positive cells in cell culture. After 15 min of calcium ionophore treatment, 48.4% of the cells displayed PS exposure on their surface. When PS targeting antibody PGN632 was preincubated with the non-treated hAxl/IFNγR1 cells to mask the native PS-positive cells, the subsequently added Gas6 induced drastically reduced hAxl/IFNγR1 activation compared with the cells that were not treated with PS antibody (Figure 6B, lanes 2–4), demonstrating the requirement of PS for Axl activation. Further, to assess whether PS externalization is sufficient for TAM activation, we increased the concentration of calcium ionophore A23187 (of 1, 5, and 10 µM) and subsequently exposed cells to a subthreshold concentration of Gas6. As shown in Figure 6C, Axl activation by Gas6 was most profoundly enhanced when cells were treated with 10 µM calcium ionophore, indicating native (intrinsic) PS externalization could also induce TAM activation. These results suggest that PS-Gas6 can activate TAM-expressing cells as both cell-based ligands and possibly also in an autocrine fashion (see model in Figure 6C).

## Discussion

In the present study, we used structure-function mutagenesis and cell biological approaches to investigate molecular mechanisms for the Gas6-mediated activation of TAM receptors. Using a series of engineered TAM/IFNγR1 reporter lines, designed to detect ligand-inducible TAM receptor dimerization and postreceptor tyrosine phosphorylation (i.e., TAM receptor activation), our studies demonstrate that activation of TAM receptors by Gas6 requires γ-carboxylation of the N-terminal Gla domain. Supportive evidences include findings that; (i) non-γ-carboxylated Gas6 produced in the presence of warfarin fails to activate TAM receptors, as well as structure/activity analysis that (ii) Gla-less Gas6 fails to activate TAM receptors, and (iii) point mutations of key glutamic acid residues (E54/E55) that directly interact with PS abrogate Gas6-mediated TAM activation. By inference that γ-carboxylated Gla domains bind the surface of anionic phospholipids such as PS, our studies indicate that Gas6 requires interaction with PS and that the active ligand is a functional protein/lipid hybrid. These data are consistent with previous observations showing that Gas6 γ-carboxylation is required for the proliferation of vascular smooth muscle cells (43), for the antiapoptotic function of Gas6 on fibroblasts and endothelial cells (44), and most relevant to the present study, for the clearance of rod outer segments by RPEs (45, 46), as well as apoptotic cell clearance and synaptic pruning in the central nervous system that is mediated by TAM receptors expressed on resident microglia (23). Furthermore, elevated distribution of PS on the surface of many virus, including HIV, has been thought to function in concert with Gas6 and TAM receptors on the targeted cells to facilitate viral entry and infection effectively (47–50).

Although Gas6 requires γ-carboxylation for TAM receptor activation (dimerization), γ-carboxylation does not appear to be required for Gas6/TAM ligand/receptor interactions, as both non-carboxylated and γ-carboxylated forms of Gas6 bind to the surface of Axl/IFN-γR1 cells as shown by flow cytometry (Figures 1C,H). These data are consistent with previous reports of Lemke and colleagues showing that Gla-less Gas6 could still bind to TAM receptors (12), as well as our previous data that soluble TAM ecto-domains could also bind non-carboxylated Gas6 (10). Although it is not clear whether Gas6/TAM receptor complexes exist preformed in the absence of PS *in vivo*, or whether there is sufficient steady-state levels of extracellular PS vesicles or PS + cells to achieve the activation of Gas6, the high level of externalized PS dysregulation that occurs in stressed tissues, virally infected tissues, or in the tumor microenvironment is expected provide a strong activation platform for the Gas6/TAM axis, and a molecular rationale for why TAMs are active in the cancer microenvironment and in virally infected tissues. Similarly, it is also possible that non-γ-carboxylated Gas6 proteins, to possibly developed as cancer therapeutics, might act as ligand traps to bind and prevent subsequent TAM activation. This latter idea might offer a molecular explanation that the *in vivo* administration of warfarin could block the Gas6-Axl signaling complex in a pancreatic cancer model (29), as well as that warfarin exerts an antimetastatic action in mice *via* the Cbl-Gas6-TAM axis in NK cells (25).

The finding that Gas6 requires γ-carboxylation and PS binding for biological activity has important implications in cancer biology. This is because, unlike the case of native tissues under homeostatic conditions where externalized PS is undetectable, externalized PS is widely dysregulated and elevated in the tumor microenvironment *via* three interactive events that include (i) the high apoptotic index of highly proliferative cancers, (ii) the high metabolic and hypoxic stress of viable cancer cells, and (iii) tumor-derived exosomes (51). Mechanistically, PS externalization following apoptosis and stress is mediated by distinct molecular processes; Apoptotic cells externalize PS *via* the combined activity of Xkr8 and ATP11C (41, 52), while stress-induced PS-externalization is regulated by TMEM16 family members, in response to (transient) rise in intracellular calcium (42). Recent studies suggest that tumor exosomes are also PS-positive, likely based on the constitutive externalization of PS on native cancer cells (40). Here, we show that all of the aforementioned sources of externalized PS are able to bind γ-carboxylated Gas6 and serve as cell-based or exosome-based scaffolds to activate TAMs, although they appear to do so with different biological characteristics; Apoptotic cells and stressed cells hyperactivate Mertk and Tyro3 as cell-based opsonins with Gas6, while cell-based sources of PS do not appear to be hyperactive for Axl. On the other hand, 70–100 nm exosomes, derived from MDA-MB-231 cells are also PS positive, and these small extracellular vesicles hyperactivate all three TAMs. Whether PS + exosomes reflect a functional difference in the activation of TAMs in the tumor microenvironment is an important consideration.

Finally, while these studies support an essential role for γ-carboxylation of Gas6, and subsequent interaction with a lipid source of PS, for its ability to activate TAM receptors, we also show by using LS-MS/MS that Gas6 γ-carboxylation has a complex and likely dynamic arrangement in this post-translational modification. Both of the enzymes required for γ-carboxylation, namely GGCX and VCOR1, appear to be broadly expressed in cells, including a variety of cells that express Gas6 and contribute to the tumor microenvironment, such as monocytic cells (macrophages) and cancer cells themselves. It is possible that overexpression of these enzymes, or upregulation in their activity, could enhance the γ-carboxylation of Gas6, and by inference from the results developed here, enhance the activity of TAM ligands (model in Figure 6). Further studies examining the levels of GGCX and VCOR1 in the tumor microenvironment are meritorious, as well as developing suitable mouse models to genetically manipulate these rate-limiting enzymes for γ-carboxylation.

## Conclusion

In summary, we show here by using functional TAM reporter cell lines that both γ-carboxylation and PS binding are indispensable for Gas6 to be active as a TAM ligand. Since externalized PS is frequently dysregulated in the tumor microenvironment, the study has important implications for not only how TAMs function in efferocytosis but also how they skew immune response in cancer.

## Author Contributions

KG and SKumar designed, performed, and analyzed experiments and wrote the manuscript. SKimani, CK, and OS provided technical help in experiments. VK performed homology modeling experiments. KM provided reagents and intellectual and technical advice. PR and SVK provided intellectual and technical advice. RB conceived and coordinated the project, designed experiments, analyzed results, and wrote the manuscript. All authors reviewed the manuscript.

## Conflict of Interest Statement

The authors declare that the research was conducted in the absence of any commercial or financial relationships that could be construed as a potential conflict of interest.
